# Systematic auditing is essential to debiasing machine learning in biology

**DOI:** 10.1038/s42003-021-01674-5

**Published:** 2021-02-10

**Authors:** Fatma-Elzahraa Eid, Haitham A. Elmarakeby, Yujia Alina Chan, Nadine Fornelos, Mahmoud ElHefnawi, Eliezer M. Van Allen, Lenwood S. Heath, Kasper Lage

**Affiliations:** 1 Broad Institute of MIT and Harvard, Cambridge, MA USA; 2 Department of Systems and Computer Engineering, Al-Azhar University, Cairo, Egypt; 3 Dana-Farber Cancer Institute, Boston, MA USA; 4 Informatics and Systems Department, Division of Engineering Research, National Research Centre, Giza, Egypt; 5 Virginia Polytechnic Institute and State University, Blacksburg, VA USA; 6 Department of Surgery, Massachusetts General Hospital, Boston, MA USA; 7 Harvard Medical School, Boston, MA USA

**Keywords:** Machine learning, Proteomics

## Abstract

Biases in data used to train machine learning (ML) models can inflate their prediction performance and confound our understanding of how and what they learn. Although biases are common in biological data, systematic auditing of ML models to identify and eliminate these biases is not a common practice when applying ML in the life sciences. Here we devise a systematic, principled, and general approach to audit ML models in the life sciences. We use this auditing framework to examine biases in three ML applications of therapeutic interest and identify unrecognized biases that hinder the ML process and result in substantially reduced model performance on new datasets. Ultimately, we show that ML models tend to learn primarily from data biases when there is insufficient signal in the data to learn from. We provide detailed protocols, guidelines, and examples of code to enable tailoring of the auditing framework to other biomedical applications.

## Abstract (web summary)

Fatma-Elzahraa Eid et al. illustrate a principled approach for identifying biases that can inflate the performance of biological machine learning models. When applied to three biomedical prediction problems, they identify previously unrecognized biases and ultimately show that models are likely to learn primarily from data biases when there is insufficient learnable signal in the data.

## Introduction

Life sciences datasets have grown increasingly large and complicated. With the advent of single-cell studies and biobanks, scientists are turning to machine learning (ML) to derive meaningful interpretations of massive genomic, transcriptomic, proteomic, phenotypic, and clinical datasets. One major obstacle to the development of reliable and generalizable ML models is that auditing for biases is not a common practice in life sciences ML; in contrast, there is a large body of work in non-biological ML that addresses the identification and removal of algorithm biases^1^. Yet, biological datasets often suffer from representational biases, i.e., an imbalance or inequality in how different biological entities are represented in biological data due to evolutionary redundancies, inherent over- or under-representation of biological entities (e.g., housekeeping genes in gene expression data and interaction hubs in protein–protein interaction [PPI] data), and/or biases specific to or induced by different experimental conditions. When these biases are not identified and eliminated, the ML process can be misled such that the model learns predominantly from the biases unique to the training dataset and is not generalizable across different datasets.

When applying ML to biological datasets, it is crucial to systematically audit for biases inherent in the data. This will help us to understand how and what the model is learning in order to ensure that its predictions are based on true biological insights from the data. Here, we devised a systematic auditing framework for paired-input biological ML applications, a class of ML prediction methods, which is widely harnessed in computational biology^2^, where the goal is to predict the biological relationships between two entities.

We used this framework to identify biases that have confounded the ML process in three applications of great interest to the life sciences and biotechnology communities: PPIs, drug-target bioactivity, and MHC-peptide binding^3–5^. Ultimately, we show that ML models tend to learn primarily from data biases when there is insufficient signal in the data for the models to learn from. We provide detailed protocols, guidelines, and examples of code to enable tailoring of the auditing framework to other biomedical applications (Supplementary Notes 1 and 2).

## Results and discussion

### Protein–protein interaction predictors

Mapping PPIs is critical to understanding cellular processes, interpreting genetic data, and predicting new targets for therapeutics development. This has led to a great interest in developing PPI classifiers that learn from previously characterized interactions to infer whether a given protein pair is likely to interact based on their protein features (summarizing information used to describe proteins to inform the ML models about their characteristics from which the model should learn, e.g., amino acid physicochemical properties). In particular, the ultimate goal of PPI classifiers is the ability to predict PPIs based on nothing but protein sequence, i.e., without structural or evolutionary information. Accurate structure- and evolutionary-based PPI predictors exist, but require PPI structure characterization or evolutionary history, thereby excluding the majority of novel, less well-characterized proteins that are the targets of key interest for PPI predictors; we typically aim to predict interactions for proteins that are not characterized rather than proteins for which structural and evolutionary data already exist. Furthermore, a structure-based approach would not be easy to extrapolate to peptides that are structurally flexible. For the past two decades, these limitations have driven the demand for PPI predictors that rely on amino acid sequences alone.

### PPI predictors do not generalize suggesting unknown biases

A critical and unexplained observation regarding such sequence-based PPI classifiers is that they achieve very high and, sometimes, near-perfect performances^2,6–8^. These models use simple summarizing sequence-based features such as frequency of k-mers (amino acid combinations of k residues), which, from a biochemical and molecular biology perspective, should not be sufficient to very accurately determine physical interactions between proteins. The typically utilized feature designs, detailed in Methods, do not take into consideration which protein residues contribute to interactions or the spatial relations among residues. Therefore, a central question in the field is: what are PPI classifiers learning from simple protein sequence features such that they can predict PPIs with near perfect accuracy?

Park and Marcotte further observed that the high performance of PPI predictors is limited to scenarios where the tested protein pairs have examples of their other interactions in the training set (examples of interacting and likely non-interacting protein pairs used to train ML models to make predictions)^2^. For example, if the training dataset contains PPI examples for proteins *A* and *B* but neither *C* nor *D*, predicting for the pair (*A*,*B*) would be accurate (we call this *in-network* prediction as both proteins appear in the training PPI network), but the prediction may be less accurate for the pairs (*A*,*C*), (*A*,*D*), (*B*,*C*), (*B*,*D*), and (*C*,*D*) (*out-of-network* prediction). Based on this logic, one could intuit that the prediction for (A,B) would be the most accurate because the model was trained on how proteins A and B interact with other proteins; on the other hand, the prediction for (C,D) would be the least accurate because the model was not trained on either protein. Following this reasoning, predictions for (A,C) or (A,D) should be more accurate than that of (C,D). However, these types of predictions have been observed to be of comparably low accuracy^2^. In other words, models are unable to make accurate *out-of-network* predictions even when trained on one of the two proteins in a given interaction. This suggests that, rather than simply being unable to generalize to proteins absent in the training set, these models may not be learning biological characteristics of the training set protein sequences that are pertinent to informing PPI predictions.

### An auditing framework to examine predictor biases

**Fig. 1:**
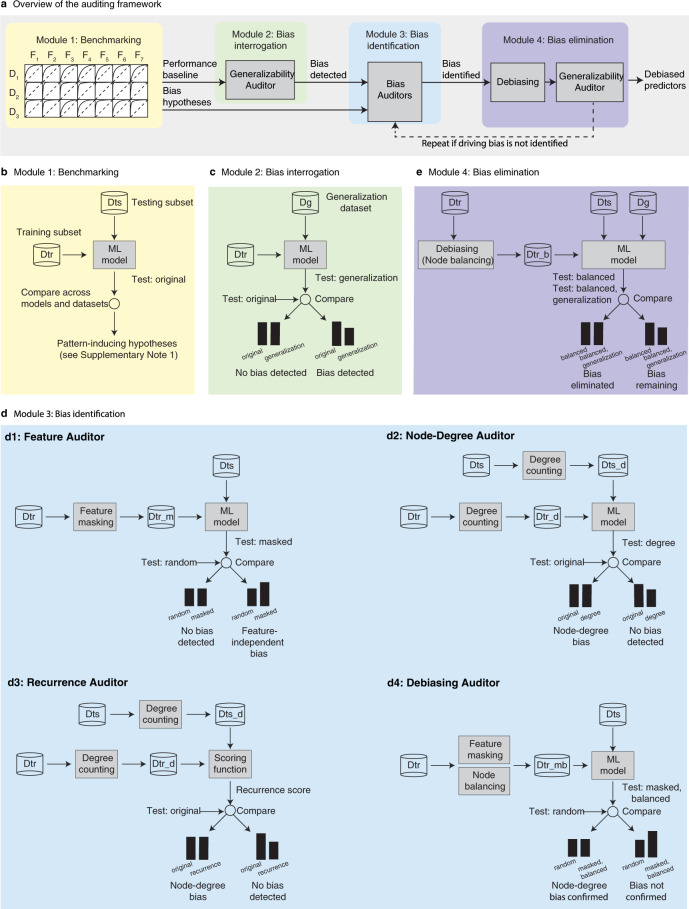
A systematic auditing framework for ML applications in biology. **a** Presentation of the four modules of the auditing framework. **b** In the Benchmarking Module 1, the ML model is trained and tested on a split dataset (Dtr and Dts, respectively) to generate a ‘Test: original’ performance for a given dataset and ML model. Performances are compared across different models and datasets to suggest bias sources that can be examined in subsequent modules as detailed in Supplementary Note 1 (Systematic Auditing Protocol). **c** The Bias Interrogation Module 2 compares the original performance of the model to its performance when tested on an independent dataset, Generalization dataset (Dg), to detect a bias. **d** The Bias Identification Module 3 modifies the data or model used in training and compares the modified with the original performances to reject or confirm the formulated bias hypotheses. The auditors here are examples of the bias identification process in paired-input problems. In the Feature Auditor in d1, the model is trained on the original training dataset but with the features masked (Dtr_m), and tested on the original test set (Dts). The performance of Test: masked is compared to the expected random performance, Test: random, e.g., when AUC is used, the Test: random AUC is 0.5. If Test: masked significantly outperforms Test: random, there is likely a bias in the dataset, independent of the features, that drives the non-random performance. In the Node-Degree Auditor in d2, each interacting object in the training dataset is represented by its node degree counts in the positive and negative training datasets to constitute Dtr_d. A model is trained on Dtr_d and tested on the test set Dts_d where each object in the original Dts is represented by its node degrees in the training datasets, Dtr. The performance of Test: degree, is compared to the original performance, Test: original. If there is no significant difference, there is likely a bias related to node degree recurrence in the original dataset. The Recurrence Auditor in d3 is similar in structure to the Node-degree auditor in d2, except that the ML model is replaced by a function to score the probability of an interaction between a pair in the test set (Dts) based on the differential node degree of the pair in the positive and negative training sets (recurrence score). These are compared against the probabilities generated by the original model, Test: original. If the performance of the recurrence-based scoring function is similar to that of the original model, the model is likely learning from the node-degree bias. In the Debiasing Auditor in d4, the training dataset is debiased by removing the node degree bias (node balancing is performed) and the features are masked to create Dtr_mb. The performance of Test: masked is compared to the expected random performance, Test: random. If the model performance, Test: masked, balanced is equal to the expected random performance (Test: random; AUC of 0.5), then the node-degree imbalance is confirmed as the major bias source in this particular data-model combination. If the bias persists, i.e., the Test: masked, balanced performs better than random, there is likely another bias driving the learning process. **e** The Bias Elimination Module 4 tests the driving power of the bias identified in Module 3 by debiasing the data (or model) and testing whether the performance will generalize to independent datasets, i.e., test if the performance of the model on the testing subset after training the model on the debiased subset (Dtr_b), Test: debiased, is comparable to the performance on the generalization subset (Dg), Test: debiased, generalization.

These observations about the non-generalizability of PPI predictors led us to hypothesize that unidentified biases in the training data may be driving both the high performance of PPI predictors and the association of high performance with in-network predictions. To test this hypothesis, we devised an auditing framework specific to paired-input ML applications, composed of four main modules: benchmarking, bias interrogation, bias identification, and bias elimination (Fig. 1, Table 1 and Methods).

**Table 1:** Technical terms.

Term	Explanation
Training sets	Data examples we feed ML models to learn from.
Features	Extracted information used to describe entities to inform the ML models about their characteristics from which the models should learn.
ML generalization	Ability of ML models to perform well on datasets independent from which their training examples were sampled.
ML auditor	A system where a ML model of interest is compared to another ML model that is tailored to examine a specific hypothesis about the initial model.
ML auditing	Examining biases of ML frameworks by building ad-hoc ML auditors.
Representational bias	Imbalance or inequality in how different entities are represented in the data due to inherent or experimental conditions.
Paired-input prediction	A class of ML prediction methods where the goal is to predict the relationships between two entities. The ML models are thus trained on pairs of entities to learn their relationships.
In-network prediction	In paired-input prediction problems, the prediction for the pair (A,B) is in-network if the training data for the predictor contains relationships in which A and B are separately involved.
Out-of-network prediction	In paired-input prediction problems, the prediction for the pair (A,B) is out-of-network if the training data for the predictor does not contain relationships for A, B, or both.
AUC	Area Under an ROC (receiver operating characteristic) curve is a classification quality measure where an AUC of 1 represents perfect prediction performance and an AUC of 0.5 indicates random prediction.

### Benchmarking seven PPI classifiers

In the first benchmarking module, we benchmarked classifiers on different datasets to establish a baseline performance for subsequent comparisons and to identify performance patterns suggestive of data biases (Fig. 1b). We selected seven prominent PPI classifiers, which we refer to as F1–F7 in this work, representing a variety of ML algorithms and diverse protein feature descriptors. F1–F5 correspond to five representative methods used in the 2012 Park and Marcotte study;^2^ F6 is a sequence-based domain profile method that we introduced to increase the diversity of the examined feature extraction methods; and F7 is a deep learning-based PPI classifier. Details of these classifiers can be found in Methods.

The performances of F1–F7 were benchmarked on two curated PPI datasets, D1^2^ and D2^6^, which are widely used to develop and test PPI classifiers; and D3^9^, a high-quality experimental dataset (Methods). All three datasets involve human proteins and are highly relevant for the development of therapeutics. Classifiers were trained on subsets of a specific dataset, e.g., protein pairs (A,B) and (X,Y), and tested on non-overlapping in-network subsets of the same dataset, e.g. (A,X), (B,X), (A,Y), and (B,Y). Importantly, we did not include out-of-network prediction testing because PPI classifiers are already demonstrated to not generalize to out-of-network predictions^2^. Furthermore, our ultimate concern is whether in-network performance is generalizable across different datasets. As anticipated, the best benchmarking performance across all classifiers was high with an average area under the curve (AUC, a classification quality measure where an AUC of 1 represents perfect prediction performance and an AUC of 0.5 indicates random prediction) of 0.83, 0.99, and 0.92 for D1, D2, and D3, respectively (Methods and Supplementary Data 1). The performances that we measured are similar to the published performances of F1–F7, indicating the correct implementation of the classifiers.

### Robust biological ML models should generalize to independent datasets

In the second module, we built a Generalizability Auditor (an auditor is a system where a ML model of interest is compared to another ML model that is tailored to examine a specific hypothesis) to assess the ability of each classifier to generalize across independent datasets (Fig. 1c). Once again, we focused on the generalization, not for new proteins (out-of-network predictions), but for proteins with PPI examples in training (in-network predictions) sampled from different datasets. If the PPI predictor indeed learns how to accurately predict PPIs in which both proteins of a given pair have examples in training, we should observe the same high performance in the two testing scenarios: benchmarking (module one) versus independent dataset testing (Module 2). Different datasets can be context-specific, which may degrade the performance observed for in-network predictions sampled from a different dataset. However, a considerable fraction of PPIs are shared across human cells and PPIs are governed by universal principles that ML models are supposed to learn. Thus, a considerable loss of prediction power when testing in-network examples sampled from a different dataset will indicate that the predictor is predominantly learning from dataset-specific biases and is therefore not generalizable across datasets. In such cases, the bias is not empowering predictions, but rather misleading the ML process so that it does not discern the true principles that determine PPIs, even within a given context or network.

Our Generalizability Auditor examines how F1–F7 perform on a dataset independent from that used for training but only contains in-network PPI examples: we used subsets of D1 and D2 for training and in-network subsets of D3 for independent testing (Methods). In the absence of bias, the AUCs of the Generalizability Auditor and the benchmarking in the first module should be comparable. In contrast, we observed a considerable difference: AUCs of the Generalizability Auditor are noticeably lower by an average of 0.14 and 0.33 for the classifiers trained on D1 and D2, respectively (Supplementary Fig. 1), suggesting that dataset-specific biases may be confounding the learning process and inflating the performance of F1–F7.

### An auditing cycle testing various hypotheses about biases pinpoints bias source(s)

**Fig. 2:**
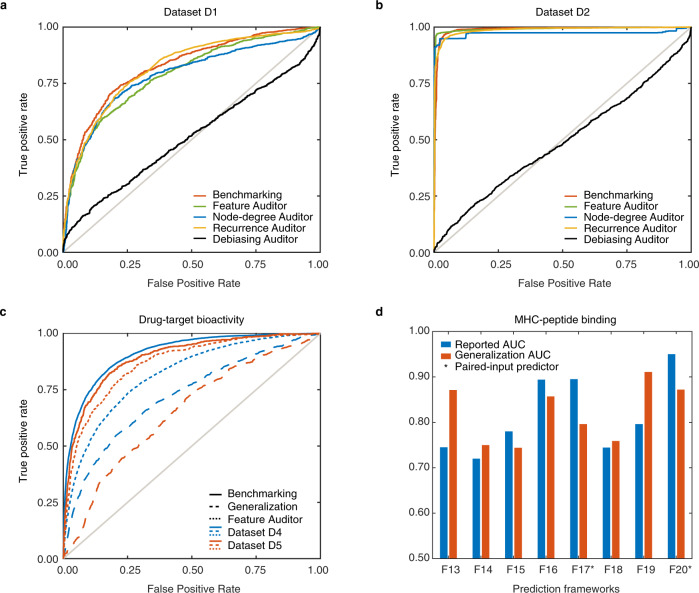
Auditing three paired-input applications of therapeutic interest. **a** The performance of the best performing PPI classifier, F1, on dataset D1 for benchmarking and the four auditors. In the Feature Auditor, protein features are masked; yet, classifier performance is retained. In the Node-degree and Recurrence Auditors, proteins are represented solely by their node degrees in the positive or negative training dataset or by their differential node degree between the positive and negative training examples; yet, without protein features, classifier performance informed by node degree or protein recurrence alone is retained. **b** The performance of the best performing PPI classifier, F5, on dataset D2, with similar observations as with Fig. 2a. **c** The performance of the optimized drug-target predictor F8 on datasets D4 and D5 in the benchmarking (module 1), generalization (module 2), and bias identification (module 3, Feature Auditor). **d** The reported (module 1) and generalization (module 2) performances of MHC-peptide binding predictors F13–F20. Asterisks denote the two paired-input predictors, F17 and F20; the other predictors are single-input.

In our third module, we established and followed a principled cycle of steps to iteratively formulate and test hypotheses regarding potential biases via hypothesis-specific auditors (Fig. 1d). Each auditor is an auxiliary ML model that is designed to assess a specific hypothesis about an ML model of interest, i.e., F1–F7 (Methods). We started by testing the hypothesis that each PPI classifier is learning solely from protein features, as it is designed to, by conceiving a Feature Auditor that masks all protein features simultaneously by replacing protein sequences with random amino acid sequences to prevent the ML models from extracting knowledge from those features (Methods). When the protein features are masked, the auditor performance should become random. Yet, we found that the benchmarking performance of each PPI classifier was largely retained regardless of the underlying ML classifier, hyperparameter values, training dataset, or protein features: average differences in AUC of −0.01, 0.00, and −0.01 were observed for D1, D2, and D3, respectively (Fig. 2a, b and Supplementary Data 1), suggesting that F1–F7 are learning from biases rather than from protein features.

We next hypothesized that protein recurrence in the training data was inflating the performance. This hypothesis was inspired by a suggestion by Park and Marcotte that if protein *x* has more positive training interaction examples, models will learn to predict pairs involving *x* as interacting, which they note often turn out to be experimentally true (see the Supplementary Discussion of Park and Marcotte 2012)^2,10^. We sought to verify this suggestion and understand the extent to which protein differential recurrence in the positive versus negative training dataset for each of the two proteins in a pair dictates predictions. This is important because predictors should be learning how protein sequence guides PPIs and not from the protein frequency in the training dataset.

To test this hypothesis, we built a Node-degree Auditor in which each protein is solely represented by its node degrees in the positive and negative PPI training examples (Methods). The performance using the Node-degree Auditor was highly similar to the best benchmarking performance across all classifiers for each dataset: differences in AUC of 0.03, 0.01, and 0.05 were observed for D1, D2, and D3, respectively (Fig. 2a, b). These results confirmed that protein recurrence is largely informing and inflating the performance of F1–F7.

Based on the observations from the Feature Auditor and Node-degree Auditor—that F1–F7 are not learning from protein features but rather from protein recurrence in the training datasets—we built a Recurrence Auditor that uses the differential node degrees of a given protein between the positive and negative training examples as the sole information to estimate the probability of PPIs (a mathematical function, not a ML classifier, that maps the combined differential recurrence of a protein pair to PPI probability). We found that the performance using the Recurrence Auditor is similar to the best benchmarking performance across all classifiers for each dataset: differences in AUC of 0.00, 0.04, and 0.06 were observed for D1, D2, and D3, respectively (Fig. 2a, b and Methods). This confirmed that the performance of F1–F7 is primarily determined by the difference in protein recurrence in the positive versus negative training dataset (Supplementary Discussion).

Finally, we implemented a Debiasing Auditor that accounts for protein node degree bias by removing differential recurrence (implemented by forcing each protein to have an equal node degree in the positive and negative training examples) while protein features are masked. If differential recurrence is a strong performance driver, this auditor should exhibit near-random performance. As expected, the predictions were effectively randomized across all combinations of classifiers, hyperparameter values, training datasets, and protein features: AUC averages of 0.50, 0.53, and 0.49 were observed for D1, D2, and D3, respectively (Fig. 2a, b and Supplementary Data 1), confirming the hypothesis that differential recurrence is strongly driving PPI classifier performance. In other words, F1–F7 are not learning from protein features to predict PPIs, but from the bias inherent in the training datasets to predict PPIs.

### Assessing performance after bias removal

In the final module, we removed the biases identified in the third module and used the Generalizability Auditor (similar to the auditor in Module 2 but with different inputs) to assess how the classifiers generalize to independent datasets after debiasing (Fig. 1e). If training dataset biases have been removed, the benchmarking performance (from Module 1, Supplementary Data 1) and the performance of the Generalizability Auditor should be similar. We applied the Generalizability Auditor to F1–F7 with debiased training subsets of D1 and D2, distinct in-network benchmarking subsets of D1 and D2, and in-network subsets of D3 for independent testing (Methods). As predicted, this improved the PPI classifier generalizability: average differences in AUC between the benchmarking and independent testing performances of 0.06 and 0.03 were observed for D1 and D2, respectively, compared to 0.14 and 0.33 in the first module (Supplementary Fig. 1). However, the overall generalizability performance was low, indicating that the PPI predictors still did not learn enough to accurately predict PPIs even after the bias was removed.

### Extending the framework to other paired-input applications

To illustrate the broad applicability of our auditing framework in general and the applicability of the developed auditors to other paired-input applications, we adapted the auditing framework to two additional applications of important therapeutic interest: predictions of drug-target bioactivity and MHC-peptide binding. For drug-target bioactivity prediction, we examined five predictors: three classification and two regression frameworks, F8–F12, on two datasets, D4 and D5 (Methods). Once again, the drug-target predictors did not generalize as well as their benchmarking performance (Supplementary Data 2 and Fig. 2c). Although these predictors are not immune to node differential recurrence bias, they are not impacted to the same extent as the examined PPI predictors. Notably, the F8 predictor was less impacted when drug/protein features were masked in D4 as compared to D5; F8 was less generalizable when trained on D5 compared to D4. This indicated that the dataset D4 has relatively more biological signal and less bias compared to D5 for predictor F8 to learn from. The extent to which a biological dataset is biased can be influenced by numerous factors. For example, alongside the presence of “promiscuous” proteins that bind to many drugs or peptides, the size of the dataset and the experimental assay utilized to collect the dataset can greatly influence bias.

For MHC-peptide predictions, we considered eight predictors, F13–F20 (Methods). F17 and F20 are less generalizable compared to their reported benchmarking performance while the other predictors generalize considerably well (the generalized performances of F13 and F19 even exceed their respective benchmarking performances), even when the test examples are out-of-network (Fig. 2d). F17 and F20 utilize an explicit paired-input setting (similar to PPI predictors) in contrast to the remaining predictors that built separate models for each MHC allele, i.e., a set of single-input models for each predictor instead of a single paired-input model (Methods). Overall, the majority of drug-target and MHC-peptide predictors generalized in a non-random fashion, suggesting that they learnt from their input features in a more biologically meaningful way compared to the examined PPI predictors.

## Conclusions

When there is insufficient signal in the training data representation, ML models could learn primarily from representational biases in the training data. This appears to predominantly influence paired-input ML applications and can be misleading if not illuminated through auditing. We have provided detailed guidelines, tutorials, and use cases on how to tailor the auditing framework to other biological ML applications (Supplementary Notes 1 and 2), as well as code, resources, and data that can be used to rerun or reposition the auditing framework described in this article (Methods and GitHub repository: https://github.com/Elzahraa/AuditingBiologicalML). We recommend that scientists who are applying ML to biological applications help to build a community-wide stance on the systematic auditing of ML models for biases. Being cognizant of the biases that fuel the predictions of each ML model will inform their application to new datasets and clarify whether the model has truly learned from governing biological principles.

## Methods

### Datasets

D1, a curated dataset, contains 24,718 positive protein–protein interaction (PPI) examples among 7033 human proteins that share at most 40% sequence identity^2^. D1 follows the random negative sampling scheme, which is the most commonly utilized for negative training in PPI classification frameworks: negative PPI examples are generated by randomly pairing proteins not reported to interact in the dataset. D2, another curated dataset, has a predefined pool of negative examples generated by pairing proteins (from the positive example pool) that do not colocalize in the same subcellular compartment. The negative and positive PPI examples in D2 number 36,320 each, among 10,336 human proteins^6^. D3, available at http://interactome.dfci.harvard.edu/H_sapiens/host.php, is a set of 15,473 PPIs among 4569 human proteins identified using a high-quality all-versus-all Y2H system such that pairs not identified as positive PPIs can be considered experimentally negative^9^. Here, each dataset is split into 10 rounds of training, validation, and test sets. Positive and negative examples are of equal count throughout the entire study to avoid class imbalance. The testing is limited to in-network test sets throughout the study, i.e., proteins in the testing sets must have examples of their other interactions in the corresponding training sets because PPI predictors do not generalize to out-of-network predictions where one or the two proteins of a test pair has no examples of their other interactions in the training sets.

One well-appreciated challenge in the development of PPI predictors is the absence of gold standard negative training examples. This is because biological studies typically verify positive PPI examples, but do not determine the absence of interactions between given proteins. D1 sought to eschew this problem by calling randomly paired proteins as negative PPIs because the majority of proteins are not expected to interact with each other. This approach is the most widely used in PPI prediction data preparation. D2 adopted a different approach by randomly calling PPIs between proteins from different cellular locations as negative because proteins in different locations are not expected to interact with each other generally. D3 considered PPIs not identified in their Y2H screen to be negative examples. However, all three approaches suffer from their own unique caveats. Unlike positive PPI examples, negative PPI examples require much more validation to conclusively determine that a particular pair of proteins is non-interacting.

### PPI classification frameworks

The utilized PPI prediction frameworks, F1–F7, all use amino acid sequences as their sole source of protein features but vary in their feature designs and machine learning (ML) models. They were selected based on their reported high performance, popularity, and diversity covering common approaches in PPI classification. F1–F5 correspond to five representative methods used in the 2012 Park and Marcotte study^2^. In F1^11^, a signature molecular descriptor^12–14^ represents each protein by the frequencies of amino acids in 3-mer combinations. F3 categorizes the 20 amino acids into seven groups according to their physicochemical properties;^7^ each protein sequence is then represented by the frequency of each possible 3-mer combination of these groups. F4^15^ accounts for the amino acid neighborhood context via an autocorrelation descriptor of seven physicochemical properties of each amino acid. F2^16^, F5^2^, and F7^8^ use the same protein descriptors as in F1, F4, and F3, respectively. We introduced F6, a sequence-based domain profile method, to increase the diversity of the examined feature extraction methods. In F6, each protein is represented by its domain profile, generated by scoring the alignment of the protein amino acid sequence to the HMM profiles of 16,712 domains downloaded from Pfam in January 2018.

F1–F4 and F6 use support vector machines (SVMs) with different kernels. F6 uses the kernel in Equation [1] where (A,B) and (C,D) are two pairs of proteins. F5 utilizes a random forest classifier while F7 utilizes a stacked autoencoder (a deep learning representational learning model). Further details of F1–F5 and F7 can be found in their respective publications^2,6–8,11,15,16^. We implemented the seven methods using MATLAB^R^ and paired it with LibSVM library^17^ for the SVM methods (F1–F4 and F6).

1 $$K\left( {\left( {A,\;B} \right),\;\left( {C,\;D} \right)} \right) = \exp \left( { - \gamma \left[ {\min \left( {||A - C||^2.||B - D||^2,\;||A - D||^2.||B - C||^2} \right)} \right]} \right)$$

### Benchmarking

D1-D3 were used for benchmarking the performance of the seven PPI classifiers, F1–F7. Model optimization was performed over 10–20 different combinations of the ML model hyperparameter values. Overall, we have examined 100 different models. Each was trained and tested on the 10 splits of each dataset, totaling 3000 experiments. We did not limit benchmarking to the models with optimized hyperparameter values. The best performing models were noted for further comparisons.

### Auditors

In AI auditing, an auxiliary ML model is designed to systematically examine bias hypotheses of an ML model of interest (main model) or its training data using the latter model input and output; a performance measure comparing the two models is defined to assess the hypotheses^1^.

#### Generalizability auditors

Two generalizability auditors, G1 and G2, were used to assess the in-network performance generalization to independent datasets before intervention (bias interrogation step) and after debiasing (bias elimination step), respectively. The main models in both auditors are the seven models optimized for D1 and D2. However, the training data for G1 is the one used for benchmarking whereas the training data for G2 is debiased first as explained in the *Debiasing Auditor* below. The test examples for the main models are subsets of D1 and D2 that satisfy the in-network performance criteria as in regular benchmarking. The auxiliary models are the same as the main models. However, the test examples for the auxiliary models in both auditors are sampled from D3 such that they satisfy the in-network test criteria for each training round. The generalizability gap was used to assess the difference in performance: the generalizability gap is the difference between the reported performance on the benchmarking test datasets (main model) and the performance on independent datasets (auxiliary model). When the gap is large, this implies that the main model does not generalize well.

#### Feature auditor (A1)

The PPI classifier of interest is used as both the main model and the auxiliary model with the same hyperparameter values. In the auxiliary model, a random feature vector is constructed for each protein and used throughout the auditing experiment: each protein sequence is replaced with a random amino acid sequence before extracting the protein features. The difference in AUC of the auxiliary model to a random classifier performance (AUC ~0.5) is used to assess the randomization efficiency.

#### Node-degree (A2) and recurrence auditors (A3)

The main model in both auditors is the best performing model for each benchmarking dataset (a single model per dataset). In A2, the auxiliary model is a simple (random forest) PPI classifier trained on the node degree of each protein in the positive and negative training networks (each PPI example is thus represented by a feature vector of length four). In A3, the auxiliary model is not an ML model but a scoring function that compares the summation of the node degrees of the protein pair in the positive and negative training networks. For protein pair A-B, whose positive and negative node degrees in the training data are (A^+^, B^+^) and (A^-^, B^-^), respectively, the score (interaction probability of the pair) can be described as in Equation [2]. The auxiliary models in both cases were evaluated on the 10 splits of each dataset and the quality of replication was assessed by the AUC decrease relative to the AUC obtained for the main model. In A2 and A3, there is one auditor for each dataset such that performance is compared to the best performing PPI classifier for that dataset.

2 $$Score\left( {A,\;B} \right) = \frac{{A^ + + B^ + }}{{A^ + + B^ + + A^ - + B^ - }}$$

#### Debiasing auditor

The main model is the same as in A1. For the auxiliary model, the negative examples of each data split are restricted such that each protein in the training contributes an equal number of positive and negative training examples according to the balanced sampling technique described by Yu et al.^18^, which presents an unbiased alternative for random sampling. However, there was insufficient evidence to support the approach’s utility in removing bias^2,10,18–21^). Other debiasing strategies for ML models or training data can be designed as needed.

The features of each protein were replaced by random numbers as in A1. For the asymmetric classifiers, i.e., F4, F5, and F7, which treat a pair [A,B] differently from [B,A], we accounted for interaction symmetricity (non-directionality of protein interactions) by utilizing the debiased sets prepared for the symmetric learners and representing each interaction [A,B] in the training data with the pairs [A,B] and [B,A].

Removing the representational bias was impractical for D2 as only 1294 out of 2181 proteins in the negative example pool are shared with the positive pool, which has 9,449 proteins. As the original negative examples were created by pairing non-co-localized proteins, we downloaded the GO localization annotation^22^ of the proteins in D2 and split them into the following high-level co-localization groups: cytoplasm, nucleus, mitochondria, and exocytic. We constructed the negative pool by pairing all proteins that do not share a subcellular location (the same way that negative sampling was originally performed for D2) and randomly selected a subset that balances each positive training set.

Throughout the experiments, the positive and negative training example counts remain equal and the test sets remain the same as in the benchmarking data splits. The randomization efficiency is assessed as in A1.

### Drug-target bioactivity prediction auditing

We considered five drug-target bioactivity prediction frameworks that predict whether (classification mode) and how strongly (regression mode) a drug can bind to a human protein target. All regression predictors are set up to predict the bioactivity response pK_*d*_ (-log10 of the equilibrium dissociation constant K_*d*_) while the classification models are set up to predict the binary binding status with pK_*d*_ = 6.3 (corresponds to 500 nM K_*d*_) used as the standard threshold for classification^23^. AUC is used to assess classifier performances while R^2^ is used for regression models. We utilized two widely used datasets in drug-target bioactivity research: the Metz dataset^24^ and a subset of the Drug Target Commons (DTC) dataset^25^, denoted here as D4 and D5, respectively. D4 and D5 consist of 107,791 and 26,634 data points measured for the bioactivity of 1497 drugs with 172 targets and 4210 drugs with 599 targets, respectively.

The first predictor is a classic drug-target bioactivity predictor^26^ that utilizes random forest models, representing drugs with their daylight fingerprints and targets with their CTD descriptor values (Composition-Transition-Distribution standard descriptors). We reimplemented the predictor for the lack of code availability and utilized it in two modes: classification mode as F8 and regression mode as F9. The second drug-target bioactivity predictor, KronRLS^27^, was used in classification mode as F10 and in regression mode as F11. KronRLS represents drug and target features in a kernalized form: Smith–Waterman (SW) score for target sequences; 2D and 3D Tanimoto coefficients for the structural fingerprints of the drugs. KronRLS imputes the missing values in the drug-target all-versus-all matrix and uses the imputed values for training (but not for testing) utilizing the Kronecker RLS model^28^. We used the published code available for KronRLS and modified it to avoid the class imbalance problem. We changed the classification threshold and the evaluation criteria as described above. The last predictor is a recent deep learning-based classifier: DeepConv-DTI^29^, a convolutional neural network classification model that processes target amino acid sequences directly and uses Morgan fingerprint as drug features.

The in-network performance for each framework, F8–F12, on D4 and D5 is used as the benchmarking performance (module 1) while the out-of-network performance (where both the drug and target in a test pair do not have examples of their other measurements in the training dataset) is used as the generalization performance (module 2). To remove the potential node-degree bias, we need to apply the balanced sampling discussed in the PPI prediction auditing. However, it was not feasible because the training datasets act as sparse bipartite graphs in the classification mode and have continuous output values in the regression mode. There are no distinct classes to balance the node degrees between them.

### MHC-peptide binding prediction auditing

We considered a set of eight well known predictors of MHC class I and class II binding peptides, F13–F20, that are recently benchmarked in the 2018 Merck study:^30^ SMM-align^31^, Comblib^32^, MHCflurry^32,33^, SMM^PMBEC^ ^34^, PickPocket^35^, TEPITOPE^36,37^, NN-align^38^, and NetMHCpan-4^39^. Testing MHC-peptide binding predictors is generally performed in the out-of-network prediction mode, where the MHC allele in a test pair has examples of its binding peptides in the training set but the peptide in that test pair is novel. To assess whether these models are biased using the *Generalizability Auditor*, we compared their reported performances in their respective publications (module 1, benchmarking) with their performances on an independent dataset from the Merck study. We examined the architecture of the predictors in their respective publications and found that only PickPocket and NetMHCpan4 utilize paired-input settings. The two predictors represent the MHC alleles in terms of the amino acid sequence of their structurally identified pockets. The auditing process was stopped after module 2 as no noticeable bias was evident for the two paired-input models; the six other models bypass node-degree bias by design and generalize well.

### Reporting summary

Further information on research design is available in the Nature Research Reporting Summary linked to this article.

## Supplementary information

Supplementary Information

Description of Supplementary Files

Supplementary Data 1

Supplementary Data 2

Reporting Summary

## Data Availability

Five datasets (D1-D3, protein–protein interactions [PPI] [PMID: 23223166; 20698572; 25416956]; D4-D5, drug-target activity [PMID: 21336281; 29276046]) and twenty classifiers (F1–F7, PPI classification [PMID: 15319262; 18269702; 17360525; 18390576; 23223166; 28545462]; F8–F12, drug-target bioactivity prediction [PMID: 23910962; 24723570; 31199797]; F13–F20, MHC-peptide binding prediction [PMID: 17608956; 15868141; 29960884; 19948066; 19297351; 10385319; 22383964; 19765293; 28978689]) used in this study are described and referenced in the Methods.

## Code availability

Scripts used in this study, tutorials, and examples of the auditing application process are described in the Methods and Supplementary Notes and are available from: https://github.com/Elzahraa/AuditingBiologicalML.
