# Supplementary material for: Systematic auditing is essential to debiasing machine learning in biology
Source: Commun Biol. 2021 Feb 10;4:183. doi: 10.1038/s42003-021-01674-5 (PMC7876113; doi:10.1038/s42003-021-01674-5)
Supplement: Supplementary file 1 — Supplementary Information [file 42003_2021_1674_MOESM1_ESM.pdf]

Supplementary Information

Systematic auditing is essential to debiasing machine learning in biology

Fatma-Elzahraa Eid[1,2]*, Haitham Elmarakeby[1,2,3], Yujia Alina Chan[1], Nadine Fornelos[1], Mahmoud ElHefnawi[4], Eli Van Allen[3], Lenwood S. Heath[5], Kasper Lage[1,6,7]*

[1]Broad Institute of MIT and Harvard, Cambridge, MA, USA.

[2]Al-Azhar University, Department of Systems and Computer Engineering, Cairo, Egypt.

[3]Dana-Farber Cancer Institute, Boston, MA, USA.

[4]The National Research Centre, Giza, Egypt.

[5]Virginia Polytechnic Institute and State University, Blacksburg, VA, USA.

[6]Massachusetts General Hospital, Department of Surgery, Boston, MA, USA.

[7]Harvard Medical School, Boston, MA, USA.

Supplementary Discussion

Generalizability of PPI classifiers. PPI classifiers are known to have poor universal generalization; a PPI classifier trained on interactions between proteins A, B, C, D, E, and F will achieve noticeably higher performance predicting new interactions between these proteins (in-network prediction) than interactions involving proteins with no examples in the training (*e.g.,* A-X, B-Y, or X-Y; out-of-network prediction) [1,2]. As a potential explanation for the high in-network performance, Park and Marcotte suggested that in-network predictions may benefit from node degree imbalances between the positive and negative training networks [3].

In this study, we examine whether the high in-network performance of PPI classifiers reflects true learning of protein features as Park and Marcotte suggested. If the classifiers truly learnt from protein features to make PPI predictions, they should generalize well to independent in-network test examples; the in-network performance should not change dramatically when the in-network test PPI examples, sampled from an independent dataset, only involve proteins that are also present in the training dataset. For the purposes of testing this form of in-network generalizability, we ensured that each protein had distinct PPIs that were present in the training examples and test examples. In other words, we would only train a classifier on a subset of the known positive and negative interactions, and reserve a non-overlapping in-network subset of the known interactions for testing. The in-network test examples from the independent dataset were curated so that they did not include PPIs identical to those in the utilized subset of the training examples.

Indeed, our auditing framework demonstrated that classifiers do not generalize to in-network test examples from independent datasets, effectively demonstrating that the high classifier performance is not solely driven by true learning of protein features. Instead, we demonstrate that the PPI classifiers learn the node degree bias of the training examples and use this as the sole information to make predictions. The node degree of a given protein changes between datasets in contrast to the protein features of a given protein that do not change regardless of the dataset. Hence, a classifier that predominantly learns from node degrees in a training dataset is incapable of accurately predicting interactions between those proteins in an independent dataset with a different node degree bias. This illustrates the need for auditing frameworks, such as the one we

designed, to systematically identify unexpected biases that impact the generalizability of classifiers.

Node degree bias. Node degree bias drives classifier performance as follows: All interactions (e.g., A-X, B-Y) that involve either protein A or B share an identical half of their feature vectors to that of the A-B interaction. For example, if the training dataset has pairs A-B and C-D as positive and E-F and A-G as negative training examples, the A-C interaction is evaluated as being more similar to two positive training pairs and only one negative pair, resulting in a ~⅔ probability of being a positive interaction. The classifier function can thus be described by the scoring function in Equation [2] (**Methods**) where the predicted score is the sum of A and B's node degrees in the positive training network relative to the sum of their node degrees in both the positive and negative training networks. When we repeated the benchmarking of classifiers after removing node degree biases from the training datasets (balanced sampling was adopted as opposed to random sampling), classifier performance dropped significantly: the average drop in AUC was 0.19, 0.35, and 0.23 for D1, D2, and D3, respectively (**Supplementary Data 1**). This suggests that existing classifiers can be improved upon to derive more accurate PPI predictions.

Supplementary Note 1

Protocol for auditing biological Machine learning applications.

Introduction. Machine learning (ML) applications in biology can suffer from unexpected biases that can inflate performance, and, if overlooked, propagate throughout the scientific community through years of subsequent research efforts. Increased attention is being paid to common biases in well-established and heavily-researched ML application fields such as image, speech, and text processing[4–6]. However, applying ML to biological data is more challenging for two main reasons: First, biological data is prone to numerous biases caused by measurement errors, different experimental conditions, and natural biological fluctuations. Second, the application of ML to biological data is still an emerging field, and one where biases have not been systematically addressed thus far.

Here, we present a general auditing framework protocol that systematically identifies biases in biological ML applications and is not limited to a certain application type or bias. The Main Text discusses in detail how to apply this auditing protocol to three examples of biological ML prediction applications that are of the type 'paired-input'. In this document, we provide additional details of the four modules (benchmarking, bias interrogation, bias identification, and bias elimination) to enable users to apply this protocol more widely to other biological ML applications.

Advantages and limitations of the protocol. This auditing protocol is intended to guide users through a systematic examination of their biological ML models to identify biases and the sources of these biases by using AI auditors. Since the ML framework, architecture, and biological data type used for each ML framework can differ, biases will require custom auditors to be detected. For that reason, it is not possible to provide a generalizable software application that works for all types of ML frameworks and biological data. However, the underlying principles of the auditing framework provided below are fully generalizable, and will enable the user to search and recognize biases and design AI auditors customized to specific applications. Importantly, we also provide general guidelines on how to interpret the auditing results.

Assumptions: An ML framework is a system that encompasses all the components necessary to develop an ML application starting with raw input data. Specifically, the ML framework executes data processing; feature extraction; data splitting into training and testing sets; ML model selection; model training; and model testing for performance evaluation. Below we provide a step-by-step guide for how to audit an ML framework F_1 that is trained on Biological dataset D_1.

Procedure:

Module 1: Benchmarking. The goal of benchmarking is to establish a performance baseline of the framework of interest (F_1) compared to other frameworks, which will then be utilized in Module 2 (Bias interrogation) to detect biases and then to form hypotheses about bias sources (if any are detected).

1. Set up *n* frameworks, F_2, F_3, ..., F_n, for benchmarking in addition to and different from framework F_1, using the following guidelines:
   a. If computational resources allow, the larger the *n*, the more robust and faster the auditing process can be. We suggest that 5-10 frameworks are needed in most cases.
   b. If your framework is an improvement over pre-existing frameworks, find the code for or re-implement a number of previous widely-used/ reliable/ well-established frameworks.
   c. If neither code nor sufficient information to replicate previous frameworks exist, re-implement the frameworks as good as possible and replace poorly described components with appropriate alternatives (i.e., a heuristic assumption of the details of the implementation, an arbitrary implementation, or the corresponding implementation from your own framework F_1). The goal of benchmarking in this step is not to show how well your framework performs compared to previous frameworks, but to spot the framework components that are potential bias sources (if any are detected).
   d. If your framework treats a new prediction problem where no previous frameworks exist, generate variations of your framework by adopting different implementations for the

various components of the ML framework. For example, adopt different ML models, data processing schemes, and training-test split schemes.

2. Collect m datasets, $D_2, D_3, \ldots, D_m$, independent from the one used for training and testing your framework (D_1). The larger the m, the more robust and faster the auditing process can be.

3. Withhold a subset $d_1, d_2, \ldots, d_m$, from each dataset, $D_1, D_2, \ldots, D_m$, respectively, for benchmarking. The percentage to withhold from each dataset should leave sufficient data points in d for benchmarking and sufficient data points in D for appropriately training and testing the frameworks.

4. Train the frameworks $F_1, F_2, \ldots, F_n$ on datasets $D_1, D_2, .., D_m$ with the benchmarking subsets excluded (D_1-d_1, D_2-d_2, ..., D_m-d_m). For each framework F_i, m trained predictors should be produced, $F_{i1}, F_{i2}, \ldots, F_{im}$, trained on D_1-d_1, D_2-d_2, ..., D_m-d_m, respectively.

5. Test each framework F_{ij} (F_i trained on dataset D_j-d_j) on the corresponding benchmarking subset d_j.

6. Build a benchmarking performance $m \times n$ matrix, B, where each cell B_{ij} corresponds to the performance of Framework F_{ij} (F_i trained on dataset D_j-d_j and tested on the benchmarking subset d_j).

Module 2: Bias interrogation. The goal of this module is to determine whether biases exist in the predictor performances by building a *Generalizability Auditor*.

7. Build a *Generalizability Auditor* as follows:

7.1 Identify k generalizability datasets, $D_{m+1}, D_{m+2}, \ldots, D_{m+k}$. These datasets will be used to assess the generalizability of the framework and should be independent of $D_1, D_2, \ldots, D_m$ (previously used for training and benchmarking).

7.2 For each dataset D_{m+z} (where $0 < z \leq k$), build a generalization performance matrix G_{m+z} (similar in shape to the performance matrix in Step 6), where each Framework F_{ij} (trained on dataset D_j-d_j and benchmarked on d_j) is tested on D_{m+z}.

7.3 Compare B (the benchmarking performance matrix, Step 6) with G (the generalization performance matrices, Step 7.2) to determine if your framework of interest, F_1, is biased. Biases will result in a "generalizability gap", which is defined as the difference in performance between the generalization and benchmarking performances ($G-B$, can be scaled or normalized). When biases exist, the generalizability gaps are noticeable.

8. Determine whether your framework (F_1) is biased based on the generalizability gaps identified in Step 7 as follows:

a. If the generalizability gaps are all negligible or positive (the latter meaning that the generalization performance is better than the benchmarking performance) for all versions of your framework F_1 (F_{11}, F_{12}, ..., F_{1m}) when tested on all of the generalization datasets (D_{m+1} … D_{m+k}), then F_1 does not suffer from major biases and the auditing process can be terminated here.

b. If the scenario in (*a*) applies to all except a minor set of the generalization datasets, F_1 can still be considered unbiased in this scenario and the auditing process can be terminated here.

c. If the generalizability gaps are all noticeably large for all versions of F_1 when tested on all of the generalization datasets, then F_1 is highly likely to be biased.

d. If the scenario in (*c*) applies to all except a minor set of the generalization datasets, F_1 still should be examined as a potentially biased framework.

e. If F_1 is generalizable (generalizability gap is insignificant or positive) in some cases, but not in others, there can be a bias in F_1 or the datasets used for training (D_1 … D_m).

9. If F_1 is likely to suffer from some bias (as determined in Step 8), determine whether the bias source is from the framework or associated with the training or benchmarking datasets. In the former scenario, identify the framework component(s) likely causing the bias by examining the

performance of the other frameworks F_2, F_3,..., F_n relative to F_1 to form hypotheses about the source of the bias:

a. Discard the performance matrices of the outlier generalization datasets (identified in Step 8.d), if any exist.
b. If the performance of all frameworks is generalizable, except when they are trained on a particular benchmarking dataset, this can indicate a bias associated with that dataset. In this case, F_1 is likely not biased and the auditing process can be terminated here. You should use this dataset only with caution and further examination of its source of bias is recommended should you use it in the future.
c. If the performance of some of the frameworks is not generalizable while others are, then F_1 is biased either at the data processing or the ML modeling stage. This can be further examined in Module 3 by examining each component of F_1 compared to the generalizable and non-generalizable framework groups.
d. If the performance of all other frameworks is similarly not generalizable, there can be a data bias either associated with the structure of the training datasets, the type of the input data utilized for this ML problem (as is the case with the protein-protein interaction [PPI] data in the Main Text) or the data processing schemes of the different frameworks (feature extraction, data split, data preprocessing, and so forth).

Module 3: Bias identification. The goal of this module is to form and verify hypotheses about the specific nature of the bias by building bias AI auditors.

An AI auditor is a system that tests an AI/ ML component/ model/ framework for a hypothesized bias. This is done by comparing its input and/or output to those of another AI/ML model you design (auxiliary model) to enable examining your bias hypothesis[6] (for examples of auditor designs see the Main Text and Supplementary Methods). As each bias can be unique, no single auditing system will fit all cases, biases, models, units, and frameworks. We therefore here provide guidelines so that users can audit components of their framework that are biased based on the results from Module 2 (Step 9).

10. Build a bias identification auditor, or a series of auditors, to examine the potential bias hypothesis.

a. If the framework component (data splitting, feature extraction, model training, and so forth) that is likely to introduce the bias is identified in Module 2 (Step 9), build an auditor to examine this particular component. Considering your framework as the main model, iteratively build auxiliary models by replacing the identified component in your framework with the corresponding component from the biased framework groups and then, the non-biased framework group. Rerun the benchmarking step (Module 1) for the auxiliary models and then the *Generalizability Auditor* (Module 2). Compare the generalizability performance of the auxiliary models of the biased and unbiased groups to that of F_I. If the examined framework component carries the bias source, you will likely find a pattern differentiating the two groups.

b. If the suspected framework component from Module 2 is not confirmed as a bias source in Step 10.a, perform a combinatorial search by repeating the same auditing process in 10.a with another component substituted (along with the examined one). In this case, we are testing the combined effect of two components causing the bias. Start with one additional component, then increase the number of additional components until a pattern emerges.

c. If the framework component that is likely to introduce the bias is not identified in Module 2 (Step 9.c), perform Step 10.a to systematically audit each component in framework F_I. If no single component is identified, move to Step 10.b and repeat for each framework component. Selecting a framework component to examine next should be prioritized as follows: i) if a component in F_I is shared with some of the generalizable frameworks, skip it; ii) if a component in F_I is shared with some of the generalizable and non-generalizable frameworks, skip it; iii) if a component in F_I is shared with some of the non-generalizable frameworks, but not the other framework group, audit it [see 10.a]; and iv) if a component in F_I is unique and not shared with any of the two framework groups, replace the corresponding components in the other frameworks of the two groups with this F_I component implementation and repeat Step 10.a (and Step 10.b if necessary) to build the auxiliary models in your auditor.

d. If the data type/ structure is the suspected source of bias (Step 9.d), or Steps 10.a, 10.b, and 10.c fail to identify the biased component, the final recourse is to examine the structure of the data as it can be presented to the framework(s) in a way that enables biased learning (this is the case with the PPI prediction auditing in our study, see Main Text).

To audit the structure of the data, you need to examine how differently the data are processed or presented compared to common ML practices. You then need to build an auditor to examine the property you identified. As each data type is unique and can carry different biases related to how the learning process is performed, this component requires thoughtful investigation of the data and its processing to be able to generate hypotheses about the bias. Here, it is critical to understand how each ML model processes the data, and relate this to the structure of the data, to infer how it may mislead the learning process. Once a bias hypothesis is formed, an auditor should be designed (as explained earlier throughout Step 10) to verify this hypothesis. Here are some considerations for probing data structures:

i. <u>Is there any class/ input/ output imbalance fed to the ML models?</u> For example, ML classifiers typically expect equal numbers of positive and negative training examples while ML regression models can be misled by overrepresented output values.

ii. <u>Do the ML models learn from the features only?</u> The *Feature Auditor* can help answer this question by randomizing the features and comparing the performance to that of benchmarking performance. This removes the information that can be derived from the input features by masking them with random numbers while keeping the structure of the training and testing datasets intact. Testing different hypotheses requires various approaches to randomizing features. For example, in the PPI prediction example, we randomized the features of each entity (protein) and carried those randomized features to represent the same protein during training and testing. If the performance after feature masking/randomization is not random (as was the case for the PPI predictors in the Main Text), this indicates the models learn some structure of the data.

iii. Is the data extracted from some complexes, networks, or bipartite graphs that carry a bias? Biological networks are usually not uniform, meaning that they are biased by nature. Probing this bias ensures that learning is not propagating it.

iv. Is there a common difference between the benchmarking datasets and generalization datasets? For example, if all the benchmarking data comes from curated databases and each generalization datasets comes from a single large scale experiment, batch effect and noise of mixing multiple studies of different qualities in the curated datasets can be a source of the generalizability gap. In this case, there can be no bias in the ML frameworks. Switching some members of the two dataset groups can be sufficient to audit this bias possibility.

11. Build a *Debiasing Auditor* to validate that the bias identified in Step 10 is driving the learning process.

Remove the identified bias from the data or the framework and then reapply the auditor that identified the bias to assess whether the bias no longer exists (if none of the auditors in Step 10 does this). For example, in the PPI *Debiasing Auditor* in the PPI prediction case (Supplementary Methods), the *Feature Auditor* was used to identify the bias source (node degree distribution), and a *Debiasing Auditor* was designed to remove the bias (by balancing the node degree between the positive and negative training examples). We then masked the features again (as performed in the *Feature Auditor*) to assess whether the bias has been efficiently removed.

Debiasing auditors are only necessary if the auditors in Step 10 are not sufficient to demonstrate that the identified bias is the only and true driver of the learning process. Some datasets can have biases that can be made ineffective by the way that ML models represent the data and/or perform the learning process. For example, as we showed in the Main Text, the MHC-peptide paired-input predictors are prone to node-degree bias similarly to PPI predictors. Nonetheless, they generalize well to new datasets, while the PPI predictors do not because of differences in feature representation informativity in these two distinct problems. This auditor is also helpful in identifying whether there can be additional major biases driving the ML frameworks, in which case another auditing round is necessary.

Module 4: Bias elimination. Once the bias source is identified and confirmed, this bias should be removed and the generalizability should be reassessed.

12. Debias the datasets or predictors. The same debiasing technique as the one utilized in the *Debiasing Auditor* (Step 11), or its equivalent (Step 10), should be used to debias the datasets or the framework. This should be done without applying the remaining steps in the *Debiasing Auditor*.

13. Assess generalizability after debiasing. The *Generalizability Auditor* (Step 7) should be reapplied to compare generalizability gaps before and after removing biases. Here are some guidelines to interpret the auditor results:

a. If the generalizability gap is eliminated, then the auditing is complete and the debiasing step needs to be included as part of the ML framework.
b. If the generalizability gap is not eliminated but noticeably reduced, while the performance after debiasing is acceptable, there might be other unidentified biases in the datasets or framework. In this situation, you will need to go through the auditing framework again, but with the datasets/ framework component debiased.
c. If the performance is noticeably reduced after debiasing to near-random performance, this can indicate that the bias was the sole driver of the predictors and that the design/ architecture of the framework is not adequate for the problem. This was the case with the PPI predictors examined in the Main Text; subsequent experiments suggested that this is due to improper feature extraction in all of the tested predictors F1-F7.

Anticipated results. This protocol provides detailed instructions on how to examine an ML framework applied on biological data for biases and how to remove these biases with the goal of developing reliable ML tools. The auditing steps described here will: i) verify whether or not the examined ML framework is suffering from major biases, ii) identify major biases if any exist, and iii) help remove the bias. This protocol will also point the user to biases in the datasets of interest, or suggest that the architecture design of the ML framework is unsuited for the biological problem/ data type at hand.

Supplementary Note 2

Tutorials for auditing biological Machine Learning applications

This set of tutorials provides information and step-by-step guides to systematically audit biological machine learning (ML) applications. It serves as a companion to the protocol in Supplementary Note 1.

Tutorial 1: Artificial Intelligence (AI) Auditor design

An AI auditor is a system where an auxiliary AI/ML model is tailor-made to examine a hypothesis about the ML framework of interest. There are four components to an AI auditor: main model, hypothesis, auxiliary model, and auditing metric. The auxiliary model can be (i) the same as the main model using a different input, (ii) the same as the main model using the same input, but with different components (e.g., a different feature extraction scheme, data preprocessing scheme, or ML model), (iii) a different model using the same input as the main model, or (iv) a different model using the output (and probably the input) of the main model. To enable the reader to design auditors, we describe AI auditors in a variety of different situations.

Auditor 1: ***Local Generalizability Auditor***

Main model	A black box ML framework, F, trained and tested on an arbitrary dataset D_1. Its performance is assessed as classification accuracy.
Hypothesis	F does not generalize well to a specific independent dataset D_2. The goal of this auditor is to make a quantifiable assessment for this statement.
Auxiliary model	The same as the main model with dataset D_2 as input.
Metric	Difference in accuracy is an acceptable comparison metric to determine if the main model generalizes to D_2. A threshold for accuracy difference (e.g., 0.1) is set to determine non-generalizability, i.e., if the difference in accuracy between the auxiliary model and the main model is larger than 0.1, the main model does not generalize to D_2.

Auditor 2: ***Single-component Auditor***

Main model	An ML framework, F_1, with specific components (data pre-processing, feature extraction, ML model, model training etc.), trained and tested on an arbitrary dataset, D_1. Its performance is assessed as classification accuracy.
Hypothesis	Component *i* in F_1 causes the main model to perform in a biased way, reflected by low accuracy on D_1.
Auxiliary model	The same as the main model, except that component *i* in F_1 is replaced with a different component performing the same function to construct framework F_2. For example, if the audited component is a feature extraction component, the replacement should be extracting features in a different way.
Metric	Increased accuracy is an acceptable comparative metric. A threshold for accuracy difference (e.g., 0.05), is set to determine improvement, i.e., if the accuracy of F_2 is higher than that of F_1 by more than 0.05, component *i* can be identified as a source of bias and replaced by the corresponding component in F_2 in the debiasing step.

Auditor 3: ***Multi-Accuracy Auditor***[7–9]

Main model	An ML framework, F_1, trained on D_1 and tested on a similarly distributed D_2. The performance is assessed as classification accuracy.
Hypothesis	Accuracy of F_1 is biased against minority subpopulations of D_2: $p_1, p_2, \ldots, p_k$.
Auxiliary model	The same framework as F_1 with the test data replaced with each subpopulation data ($p_1, p_2, \ldots, p_k$), one at a time, to create frameworks F_{p1}, $F_{p2}, \ldots, F_{pk}$.

Metric	A decrease in accuracy of 0.05 between F_1 and each of F_{p1}, F_{p2}, …, F_{pk} is considered significant. This test identifies the subpopulations against which F_1 is biased.

Auditor 4: ***Encoding Auditor***

Main model	A black-box encoding system, E, that compresses an object (image, sequence, text etc.) into a denser representation of smaller size.
Hypothesis	E does not encode this type of data properly.
Auxiliary model	k decoder ML models, De_1, De_2, …, De_k, with different architectures and where the input is the encoded output of E and the training target is the corresponding input to E (full object prior to encoding). The ML task of the decoders is to reconstruct the uncompressed objects.
Metric	For a large and diverse dataset, an appropriate metric tests how precisely the encoded output of E can be reconstructed into the original input using the decoders De_1, De_2, …, De_k. The normalized number of matching subobjects (pixels for images, letters for texts, items for sequences etc.) between the original and the reconstructed objects can serve as a metric for reconstruction accuracy. Let the overall reconstruction metric be the average of all reconstruction ratios for all objects in the test dataset. An 80% average reconstruction rate is set as a threshold for acceptable reconstruction. When k is sufficiently large (given the complexity of the problem) and the decoders vary and are well-designed, then E is not recommended if none of the decoders pass the acceptable reconstruction threshold.

Auditor 5: ***Single-class output Auditor***

Main model	A black box classification predictor C claimed to have a small prediction error. When C is tested on dataset D, all predictions are mostly from a

	single class c_1. There is no other dataset available and the ground truth for *D* is unknown.
Hypothesis	*C* is biased during training and possibly trained and tested on class-imbalanced data where examples in class c_1 are overrepresented. The alternative hypothesis is that c_1 is truly overrepresented in *D*.
Auxiliary model	The same classifier *C* applied to dataset *D* but the feature vectors are randomized after feature extraction. Feature randomization can be performed by randomizing the feature vector of each datapoint (row-wise), substituting each value in the feature vector by a random number within the expected range of this feature values, randomizing the values of each feature for all data points (column-wise), or randomizing the feature matrix of all test data points.
Metric	Accuracy is a reasonable metric for classification problems but in this example, the ground truth values are missing. However, if the percentage of the randomized points being classified as class c_1 is within a small margin (e.g., ∓0.05) from that obtained classifying the original data points, then this classifier *C* can suffer from class-imbalance bias during training. This possibility needs further auditing. Conversely, if the percentage of the points in class c_1 for the randomized data is close to a randomly expected value (~50% for two class classifiers), then *C* does not suffer from class imbalance and class c_1 points are overrepresented in *D*.

Auditor 6: ***Regression Flat-Output Auditor***

Main model	A black box regression predictor *R* claimed to have a small prediction error. When *R* is tested on dataset *D*, the output is nearly constant, with a small variance around a single value *v*. There is no other dataset available and the ground truth for *D* is unknown.

Hypothesis	R is biased during training, possibly with an overrepresented value near v in the training and testing sets.
Auxiliary model	The same regression model R is used but the input dataset D is represented with randomized feature vectors as described in Auditor 5.
Metric	Mean deviation (average absolute difference) from the constant value v in the prediction can be a reasonable metric. If the deviation metric of the two inputs (original and randomized) is nearly the same, then R is highly likely biased during training with an overrepresented value near v.

Tutorial 2: Auditing metric design

In AI auditors, a metric is needed to compare performances between main and auxiliary models and verify the hypothesis for which the auditor is built. In the *Generalizability Auditors* of the auditing protocol, the main and auxiliary models are the same ML framework, but each model is tested on different datasets to assess differences in performance. Comparing the performance of two models is a common auditing scenario (two similar models with different inputs or two different models with similar input) and requires careful design of the auditing metric. Comparing the performance of two models is not always straightforward. In this tutorial, we discuss the design of three auditing metrics that have wide applicability towards this aim.

Example 1: Comparison of accuracy metrics of classification models

Given the classification accuracy of the main and auxiliary models (A_{main} and A_{aux}, respectively), this example describes how to assess performance differences shown in **Tutorial Table 1**.

Tutorial Table 1*: Differences in classification accuracy between main and auxiliary models.*

Case	1	2	3	4	5	6	7	8	9
A_{main}	0.85	0.75	0.65	0.40	0.85	0.99	0.99	0.80	0.70

A_{aux}	0.75	0.65	0.55	0.30	0.40	0.98	0.90	0.81	0.90
A_{aux}–A_{main}	-0.10	-0.10	-0.10	-0.10	-0.45	-0.01	-0.09	0.01	0.20

In the first four cases, the reduction in performance is the same (0.10). Strictly in terms of classification accuracy, this decrease in performance is perceived as similar, whether stemming from a reduction in performance from 0.85 to 0.75, or from 0.40 to 0.3 (Cases 1 and 4, respectively). In this situation, the arithmetic difference between the two performances is acceptable as a comparative metric.

However, a significance threshold that takes into account the nature of the examined data is necessary. A difference of 0.01-0.05 in accuracy can be considered non-significant (negligible) for most ML models, but a difference greater than 0.1 is typically considered significant (considerable). If the prediction learning task is not considered complex (meaning that high performance is easily obtainable during training and testing), a lower threshold should be adopted. It is easier to judge the performance gaps as significant or insignificant when the difference is very large or very small (Cases 5 and 6, respectively). However, for the *Generalizability Auditors* in the auditing protocol, if all gaps are close to a marginally acceptable threshold (e.g., ∓0.05), this may reflect the absence of bias and the frameworks can be considered generalizable (Case 7).

For *Generalizability Auditors*, performance gaps are logically negative ($A_{aux} < A_{main}$) because a model trained on a subset of a dataset is expected to perform better when tested on another subset of the same dataset compared to when tested on an independent dataset. However, it is not surprising to observe small positive gaps ($A_{aux} > A_{main}$, when a generalization dataset is very similar to the training sets in terms of feature vectors (Case 8). However, a large positive value (for example >>0.1) should raise concerns and is a reason to audit the generalization dataset itself (Case 9). This should be taken into account when designing a performance metric where positive and negative performance gaps may occur. For example, in *Generalizability Auditors*, for most ML models, we can consider thresholds of -0.1 and 0.1 as significant decrease and increase in accuracy, respectively.

Example 2: Comparing AUCs for classification models

Given the area under the ROC curve (AUC) of the main and auxiliary models (AUC_{main} and AUC_{aux}, respectively), this example describes how to assess performance differences in **Tutorial Table 2**.

Very small gaps between the main and auxiliary AUC values (for example, <0.05, in Cases 1 and 2) are acceptable. However, when these differences increase (for example, >0.1, in Cases 3 and 4), the absolute value of the AUCs in question becomes important. For example, although the two cases show the same decrease in absolute AUC (-0.20), a decrease from 0.90 to 0.70 means the auxiliary model is still functional while a decrease from 0.70 to 0.50 means the auxiliary model is exhibiting random behavior (for the common case of two-class classification systems, because random behavior in terms of an AUC is 0.5).

Tutorial Table 2: *Differences in AUC between main and auxiliary models.*

Case	1	2	3	4	5
AUC_{main}	0.90	0.60	0.90	0.70	0.90
AUC_{aux}	0.87	0.57	0.70	0.50	0.50
$AUC_{aux}-AUC_{main}$	-0.03	-0.03	-0.20	-0.20	-0.4
$(AUC_{aux}-AUC_{main})/0.5$	-0.06	-0.06	-0.40	-0.40	-0.8
AUC_{aux} / AUC_{main}	0.97	0.95	0.78	0.71	0.56
$(AUC_{aux}-0.5)/(AUC_{main}-0.5)$	0.93	0.70	0.50	0.00	0.00

Collectively, we conclude that the difference in AUC is not an appropriate measure for comparing two AUC values in general and scaling or normalization is needed. Scaling the difference in AUC values by 0.5 ($(AUC_{aux}-AUC_{main})/0.5$) does not discriminate between Cases 3 and 4. Taking the ratio of the two AUC values (AUC_{aux} / AUC_{main}) seems more reasonable, but the absolute value of that ratio is not intuitive. Subtracting 0.5 from both AUC values before calculating the ratio ($(AUC_{aux}-0.5)/(AUC_{main}-0.5)$) is potentially appropriate since it scales the

ratio of the AUC values relative to the reference point of 0.5. If this last metric is selected, attention should be paid to its suitability for assessing differences in Cases 1 and 2.

Example 3: Comparing mean absolute error for regression models.

Mean absolute error (MAE) is a standard regression performance metric. MAE equals the average of the absolute difference between the expected (target or true values) and predicted values for all points in a dataset using the same regression model. Given the MAE of the main and auxiliary models (MAE_{main} and MAE_{aux}, respectively), this example describes how to assess performance differences in **Tutorial Table 3**.

Tutorial Table 3*: Differences in MAE between main and auxiliary models.*

Case	1	2	3
MAE_{main}	40	400	4,000
MAE_{aux}	30	300	3,000
$MAE_{aux} - MAE_{main}$	10	100	1,000
MAE_{aux} / MAE_{main}	0.75	0.75	0.75
$(MAE_{aux} - MAE_{main})/MAE_{main}$	0.25	0.25	0.25
$(MAE_{aux} - MAE_{main})/Mean$, $Mean = 1000$	0.01	0.1	1
$(MAE_{aux} - MAE_{main})/Mean$, $Mean = 100$	0.1	1	10
$(MAE_{aux} - MAE_{main})/(max-min)$, $max = 500$, $min = 0$	0.02	0.2	2

Unlike accuracy and AUC, MAE does not rely on a reference point to indicate bad performance. Thus, taking the difference ($MAE_{aux} - MAE_{main}$), ratio (MAE_{aux} / MAE_{main}), or scaled difference ($(MAE_{aux} - MAE_{main})/MAE_{main}$) does not quantify how good or bad the gap between the two performances is.

Thus, a normalization factor is needed to account for the magnitude of the performance difference relative to the expected range of value in this problem. For example, a regression problem where the output values are expected to be in the range of 1,000 - 10,000 can tolerate errors of 30 and 40 (Case 1), but errors in the thousands (3,000 and 4,000 in Case 3) cannot be tolerated. For that, dividing the difference by the mean of the expected output values can be a reasonable metric. Also, dividing the difference by the acceptable range is a reasonable metric if the output points are uniformly distributed across this range. Different cases need to be examined for an adopted metric in order to determine the thresholds of acceptable differences along the guidelines mentioned above.

Tutorial 3: Bias interrogation - Examining generalizability gaps for framework bias potential

Generalizability gaps are metrics used in *Generalizability Auditors* to assess the difference in performance of an ML framework when tested on an independent generalization dataset compared to its claimed performance (usually when trained and tested on subsets of the same dataset). When examining the generalizability gaps in the 'bias interrogation' step to determine if the framework of interest F_1 is biased, we first examine the generalizability gaps of all versions of F_1 (F_{11}, F_{12},...), where F_1 is trained on different datasets (D_1, D_2,...) and tested on generalizability datasets (D_{g1}, D_{g2}, …). This is equivalent to singling out the columns corresponding to framework F_1 versions from each generalization performance matrix (G1, G2, …, tested on different D_{gx}) and subtracting them from the corresponding column in the benchmarking matrix *B* (see the auditing protocol in Supplementary Note 1 for details). This tutorial explains Step 8 in the protocol.

For this tutorial, assume that the performance is measured in classification accuracy and a threshold of -5% is set as a significant decrease in accuracy in generalization performance and a 5% upper bound is set as an insignificant increase in accuracy (increase of <5% is considered insignificant).

[Scenario 1]

In *Case (a)* (**Tutorial Table 4**), consider the gaps for the generalizability datasets (D_{g1}-D_{g4}) for the first five variants of F_1 (F_{11}-F_{15}). In all cases, the gaps are smaller in size (>-5%) than the threshold of significant performance decrease or slightly better than zero, but not to a significant extent (< 5%). Such small increase in performance can happen when the generalization dataset examples belong to subpopulations that are well represented in the training data and followingly are accurately scored by the model. Overall, having all gaps within the acceptable performance range (5%> gap >-5%) strongly suggests that F_1 is unbiased and the auditing process can be terminated here.

Tutorial Table 4: *Generalizability gaps (%) for a framework of interest F_1 trained on six datasets (producing different frameworks F_{11}-F_{16}) and tested on generalizability datasets D_{g1}-D_{g5} in two cases (a) and (b).*

	Case (a)					*Case (b)*				
	D_{g1}	D_{g2}	D_{g3}	D_{g4}	D_{g5}	D_{g1}	D_{g2}	D_{g3}	D_{g4}	D_{g5}
F_{11}	-2.2	-1.4	-4.0	1.0	-5.0	-6.0	-5.4	-14.0	-6.0	-.2
F_{12}	-1.9	-0.9	-3.8	0.8	-5.1	-6.1	-6.9	-18.8	-8.8	-1.9
F_{13}	-1.8	0.0	-4.1	1.3	-6.3	-5.3	-4.8	-11.1	-9.3	-1.8
F_{14}	-2.1	1.0	-4.3	1.7	-4.5	-6.9	-9.3	-14.3	-6.7	-2.1
F_{15}	-2.3	-1.1	-3.5	2.1	-7.3	-7.4	-5.1	-13.5	-7.1	-2.3
F_{16}	-18.3	-13.5	-11.2	-9.8						

[Scenario 2] Now consider the previous situation but for generalizability datasets D_{g1}-D_{g5}. The performance of all variants of F_1 (F_{11}-F_{15}) for D_{g5} slightly exceeds the acceptable gap threshold. This can indicate that the examples of D_{g5} belong to less represented subpopulations in all the training data or that F_1 is slightly biased against these subpopulations. However, since the gaps are not much higher than the arbitrary threshold, and this situation only occurred for a single

generation dataset, it is legitimate to consider F_1 unbiased and to terminate the auditing process at this stage. However, it is recommended to look into the gaps of the other frameworks (F_2, F_3,...) for D_{g5} and to confirm that this dataset is a true outlier (see Tutorial 4). This especially holds if the number or diversity of the generalization datasets is not sufficient to ignore the gaps of F_1 variants for D_{g5}.

[Scenario 3] Now consider another scenario from **Tutorial Table 4** for all framework variants (F_{11}-F_{16}) against the first four generalization datasets (D_{g1}-D_{g4}). While all other framework varian ts have gaps within the acceptable variation range, the gaps of F_{16} are noticeably larger than the acceptable threshold for all generalization datasets. This suggests a potential bias in the training data D_6 on which the framework variant F_{16} was trained. At this point, similarly to Scenario 2, F_1 can be considered unbiased and the auditing process can be terminated here. However, it is recommended to look into the gaps of the other frameworks trained on D_6 (F_{26}, F_{36},...) and to confirm the training dataset D_6 is a true outlier.

[Scenario 4] In *Case (b)*, consider the gaps for the generalizability datasets (D_{g1}-D_{g4}). The gaps for all cases are mostly larger than (or in a few cases slightly smaller than) the threshold for a significant performance decrease. This is a straightforward signal that F_1 is biased and needs to be audited.

[Scenario 5] Now consider another situation where the gaps belong to the generalizability datasets (D_{g1}-D_{g5}). For D_{g5}, gaps of all variants of F_1 are below the significance threshold. This can indicate that the examples of D_{g5} belong to the more represented subpopulations in all the training data or that F_1 has a little bias against these subpopulations. Since this situation only occurred for a single generalization dataset, considering F_1 unbiased is not justified, and thus F_1 still needs to be audited.

Tutorial 4: Bias interrogation - identifying the source of the bias

If examining the framework generalizability gaps is inconclusive (**Tutorial 3**), it is important to co-examine the generalizability gaps of the other frameworks as well to determine whether the bias is associated with i) the framework of interest, ii) training datasets, iii) benchmarking datasets, or iv) general to all frameworks. If the bias is associated with the framework of interest, co-examining the other frameworks can point to biases in components of the frameworks that are shared as well.

In Scenario 3 of **Tutorial 3** (**Tutorial Table 4**, Case *a*), all framework variants have gaps within the acceptable variation range except F_{16}, for which all gaps exceed the acceptable threshold of decrease in performance (across all generalization datasets). This pattern suggests that the training set D_6 might be biased. To confirm this, the user should examine the gaps of the other frameworks when trained on D_6 (**Tutorial Table 5**).

Tutorial Table 5: *Generalizability gaps (%) for frameworks* F_1*-*F_5 *trained and tested on subsets of dataset* D_6*, and tested for generalization on generalization datasets* D_{g1}*-*D_{g4} *in three different cases a ,b and c.*

	Case a				*Case b*				*Case c*			
	D_{g1}	D_{g2}	D_{g3}	D_{g4}	D_{g1}	D_{g2}	D_{g3}	D_{g4}	D_{g1}	D_{g2}	D_{g3}	D_{g4}
F_{16}	-18.3	-13.5	-11.2	-9.8	-18.3	-13.5	-11.2	-9.8	-18.3	-13.5	-11.2	-9.8
F_{26}	-11.9	-7.9	-13.8	-10.8	-1.9	-0.9	-0.8	-0.1	-1.9	-0.9	-0.8	-0.1
F_{36}	-11.8	-9.0	-15.1	-11.3	-0.8	-1.0	0.3	-2.3	-0.8	-1.0	0.3	-2.3
F_{46}	-12.1	-11.0	-8.3	-13.7	-2.1	-2.1	1.0	-3.2	-2.1	-2.1	1.0	-3.2
F_{56}	-21.3	-21.1	-13.5	-7.1	-21.3	-21.1	-13.5	-7.1	-1.3	-4.1	2.3	-3.1

In *Case a* (**Tutorial Table 5**), all frameworks trained on the same dataset D_6 consistently do not generalize to any other dataset. This suggests a bias within training dataset D_6 and not the

framework of interest F_1 (as long as the same behaviour is not repeated with the other training datasets). At this stage, F_1 can be declared unbiased and the auditing process can be terminated. However, it is very useful to audit D_6 to understand what may have caused this bias, which can give more insights into the machine learning process.

In *Case b* (**Tutorial Table 5**), another framework (F_5) exhibits non-generalizability that is comp arable to F_1 for all datasets when trained on D_6, while the other frameworks do not. This can indicate that F_1 and F_5 have a shared component that is biased. In the Bias Identification module of the auditing protocol, one can identify which component is introducing the bias by comparing all components of F_1 and F_5.

In *Case c* (**Table 5**), there is no framework exhibiting non-generalizability on all datasets as observed for F_1 trained on D_6, which suggests that F_1 has a unique component that introduces the bias when trained on D_6. In the Bias Identification module of the auditing protocol, one can identify which component is introducing the bias by comparing all components of F_1 to those of the other frameworks.

If we consider a situation corresponding to Scenario 3 of Tutorial 3 (**Table 4**, Case *a*) where framework F_1 is not generalizable to any generalization dataset. The user first needs to look at the generalizability gaps of the other examined frameworks. Here are possible scenarios:

a. All other frameworks are generalizable. In this case, there must exist at least a single component of F_1 that introduces the bias. By comparing the behaviour of each component of F_1 with the corresponding components of the other frameworks, the biased component can be identified.
b. Some frameworks are not generalizable, similarly to *F1*, while the remaining ones are. In this case, the non-generalizable frameworks may share a component with *F1* or have divergent components that behave similarly and introduce analogous biases. To resolve this situation:
    i. The user must examine shared components among the non-generalizable frameworks that do not exist in the generalizable frameworks.

ii. If the biased component is not identified, there may be analogous components (between *F1* and the other non-generalizable frameworks) that behave similarly in terms of biases. This needs a deeper investigation into how each component of these behaves for each framework.

iii. Finally, if nothing is identified yet, the user will need to examine combinations of two or more components that are shared among the non-generalizable frameworks, but not among the generalizable frameworks, to account for the possibility that certain combinations of components may introduce a bias, although no individual component does this.

c. All other frameworks are similarly non-generalizable. This indicates a general bias across all frameworks.

i. First, the user needs to exclude that the generalization datasets differ in quality or processing from the training datasets. For example, the training datasets may each be the results of a single stringent experiment while the generalization datasets are from curated noisy datasets. Another example is that the two sets are processed using different pipelines with divergent assumptions.

ii. If there is no common difference between the two sets, examine shared components among all frameworks. For example, they all may share a preprocessing step that introduces the bias.

iii. If no single component is shared among all frameworks, examine components that have similar behavior among all frameworks. For example, input features can be a global sum of input entities without local features. Alternatively, all ML models may be assuming independence between input features.

iv. If no single component is identified in (ii) nor (iii), repeat the two steps assuming combinatorial effect of components by examining two components at a time.

v. If the bias source remains unidentified, there can be a bias inherited in the type of biological data utilized in this problem that all frameworks capture, such as is the case with protein-protein interaction data in the Main Text.

Supplementary Figure

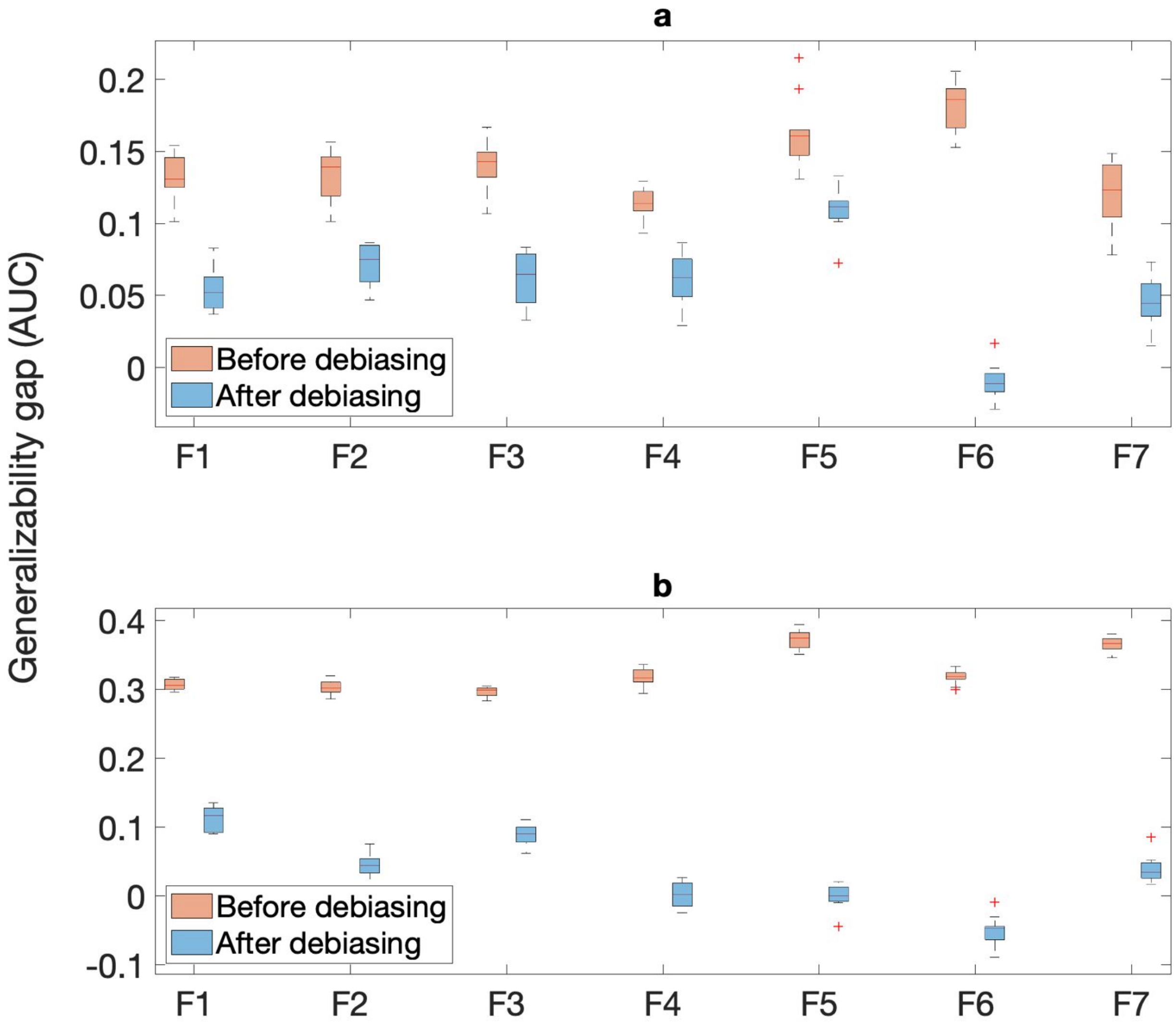

Supplementary Figure 1: Performance gap (measured by the difference in AUC between reported/ benchmarked and independent testing performances) for the seven PPI classifiers, F1-F7, when benchmarked on datasets D1 **(a)** and D2 **(b)** and tested on dataset D3 before (pink) and after (blue) removing the node degree representational bias.

References

1. Park, Y. & Marcotte, E. M. Flaws in evaluation schemes for pair-input computational predictions. *Nat. Methods* **9**, 1134–1136 (2012).
2. Hamp, T. & Rost, B. Evolutionary profiles improve protein–protein interaction prediction from sequence. *Bioinformatics* **31**, 1945–1950 (2015).
3. Park, Y. & Marcotte, E. M. Revisiting the negative example sampling problem for predicting protein–protein interactions. *Bioinformatics* **27**, 3024–3028 (2011).
4. Raji, I. D. & Buolamwini, J. Actionable Auditing. *Proceedings of the 2019 AAAI/ACM Conference on AI, Ethics, and Society - AIES '19* (2019) doi:10.1145/3306618.3314244.
5. Tannenbaum, C., Ellis, R. P., Eyssel, F., Zou, J. & Schiebinger, L. Sex and gender analysis improves science and engineering. *Nature* **575**, 137–146 (2019).
6. Zou, J. & Schiebinger, L. AI can be sexist and racist — it's time to make it fair. *Nature* vol. 559 324–326 (2018).
7. Hébert-Johnson, U., Kim, M. P., Reingold, O. & Rothblum, G. N. Multicalibration: Calibration for the (Computationally-Identifiable) Masses. in *Proc. Mach. Learn. Res. 80, 1939–1948* (2018).
8. Kearns, M., Neel, S., Roth, A. & Wu, Z. S. Preventing fairness gerrymandering: auditing and learning for subgroup fairness. in *Proc. Mach. Learn. Res. 80, 2564–2572* (2018).
9. Kim, M. P., Ghorbani, A. & Zou, J. Multiaccuracy: black-box post-processing for fairness in classification. *Preprint at https://arxiv.org/abs/1805.12317* (2018).
