# Supplementary material for: Systematic auditing is essential to debiasing machine learning in biology
Source: Commun Biol. 2021 Feb 10;4:183. doi: 10.1038/s42003-021-01674-5 (PMC7876113; doi:10.1038/s42003-021-01674-5)
Supplement: Supplementary file 2 — Description of Supplementary Files [file 42003_2021_1674_MOESM2_ESM.pdf]

Description of Additional Supplementary Files

File name: Supplementary Table 1

Description: Performance based on parameter settings in F1-7. Performance (measured in average AUC across 10 rounds) of frameworks F1-F7 on datasets D1-D3 using different parameter settings (P) in the following contexts: benchmarking (B), Feature Auditor (AF), debiased (D), and Debiasing Auditor (AD). For frameworks F1, F3, F4, and F6, C is the regularization parameter and γ is the SVM kernel coefficient. Similarly, C is the regularization parameter for F2. T and M are the number of trees and minimum leaf per node, respectively, for the random forests in F5. H and R are the hidden layer size and regularization parameter, respectively, for the autoencoder in F7.

File name: Supplementary Table 2

Description: Performance based on parameter settings in F8-12. Performance (measured in average AUC for the classification frameworks F8, F10, and F12, and in average R-squared for the regression frameworks F9 and F11) on datasets D4 and D5 using different parameter settings for F8 and F9, and the optimized parameters for F10, F11, and F12, in the following contexts: benchmarking (B), generalization (G) and Feature Auditor (AF). For F8 and F9, T is the number of trees, M is the minimum leaf per node, and N is the number of predictors per node for the random forests. The x values indicate where a model fails to operate.
